# Niche-adaptation in plant-associated *Bacteroidetes* favours specialisation in organic phosphorus mineralisation

**DOI:** 10.1038/s41396-020-00829-2

**Published:** 2020-11-30

**Authors:** Ian D. E. A. Lidbury, Chiara Borsetto, Andrew R. J. Murphy, Andrew Bottrill, Alexandra M. E. Jones, Gary D. Bending, John P. Hammond, Yin Chen, Elizabeth M. H. Wellington, David J. Scanlan

**Affiliations:** 1 School of Life Sciences, University of Warwick, Gibbet Hill Road, Coventry, UK; 2 Department of Animal and Plant Sciences, University of Sheffield, Sheffield, UK; 3 School of Agriculture, Policy, and Development, University of Reading, Earley Gate, Whiteknights, Reading, UK

**Keywords:** Transcriptomics, Bacteriology, Soil microbiology, Proteomics

## Abstract

*Bacteroidetes* are abundant pathogen-suppressing members of the plant microbiome that contribute prominently to rhizosphere phosphorus mobilisation, a frequent growth-limiting nutrient in this niche. However, the genetic traits underpinning their success in this niche remain largely unknown, particularly regarding their phosphorus acquisition strategies. By combining cultivation, multi-layered omics and biochemical analyses we first discovered that all plant-associated *Bacteroidetes* express constitutive phosphatase activity, linked to the ubiquitous possession of a unique phosphatase, PafA. For the first time, we also reveal a subset of *Bacteroidetes* outer membrane SusCD-like complexes, typically associated with carbon acquisition, and several TonB-dependent transporters, are induced during Pi-depletion. Furthermore, in response to phosphate depletion, the plant-associated *Flavobacterium* used in this study expressed many previously characterised and novel proteins targeting organic phosphorus. Collectively, these enzymes exhibited superior phosphatase activity compared to plant-associated *Pseudomonas* spp. Importantly, several of the novel low-Pi-inducible phosphatases and transporters, belong to the *Bacteroidetes* auxiliary genome and are an adaptive genomic signature of plant-associated strains. In conclusion, niche adaptation to the plant microbiome thus appears to have resulted in the acquisition of unique phosphorus scavenging loci in *Bacteroidetes*, enhancing their phosphorus acquisition capabilities. These traits may enable their success in the rhizosphere and also present exciting avenues to develop sustainable agriculture.

## Introduction

*Flavobacteriaceae* belong to the phylum *Bacteroidetes* and are dominant members of plant/soil and ocean microbiomes [1–5]. In the plant microbiome (rhizosphere, endosphere and phyllosphere), the abundance of *Flavobacteriaceae* and other members of the *Bacteroidetes* phylum is generally orders of magnitude greater than that of the surrounding bulk soil [2, 3, 6, 7]. *Flavobacteriaceae* typically represent 5–65% of the microbial community associated with various agriculturally important crops [8] and exhibit functionally important effects on plant health [8–11]. In Barley, 25% of the isolates obtained from the rhizosphere were related to *Flavobacteriaceae* and these contributed almost 50% of the potential microbial P-turnover [12]. In contrast to other important plant-associated microbiota [13–16], the genetic traits responsible for the success of *Flavobacteriaceae* in the plant microbiome, including their phosphorus acquisition strategies, have not been resolved.

In ocean and animal microbiomes, *Bacteroidetes* are key regulators of carbon cycling, and thus microbiome functioning, due to their enhanced ability to degrade complex algal and plant-derived polysaccharides [4, 5, 17]. Terrestrial *Flavobacteriaceae* have also been predicted to degrade plant-derived glycans due to the high number of glycan-specific hydrolytic enzymes encoded in their genomes [8, 18]. Polysaccharide degradation in *Bacteroidetes* utilises specialised gene clusters, termed polysaccharide utilisation loci (PULs) [19]. A prominent feature of PULs includes a two-component outer membrane (OM) transport system-termed SusCD (archetypal form, **S**tarch **u**tilisation **s**ystem). SusC is a transmembrane TonB-dependent transporter (TBDT) and SusD is the ligand-binding lipoprotein cap [20]. The specificity of the SusCD-like-complexes allows *Bacteroidetes* to grow on a variety of plant- [21, 22] algal- [23] and fungal-derived polysaccharides [24, 25]. The latter category includes the sole example of an entire PUL characterised in *Flavobacterium*.

For all biota on Earth, P is an essential macronutrient and its availability directly affects the global carbon cycle as well as governing global crop production [26–30]. Modern agriculture massively relies on the use of unsustainable chemical fertilisers, which has now created an emerging global phosphorus crisis [26, 31]. In soils, P exists in many immobilised organic and inorganic complexes whilst only a small fraction (<1%) exists in solution as orthophosphate (Pi) [32–34]. The rate plants take up labile Pi into their roots far exceeds the rate at which diffusion can replenish soil Pi surrounding the roots [32, 33]. This creates localised regions of Pi-depletion at the soil–plant interface (a region commonly referred to as the rhizosphere) [35]. Organic P (P_o_) often accounts for the majority of total P in soils and soil microorganisms play a key role in solubilising this fraction before it becomes available to plants [33, 34, 36]. However, rhizosphere microbes also contribute to the immobilisation of labile Pi, thus competing with plants for P [32, 37]. Therefore, developing a greater understanding of soil microbial P dynamics at the soil–root interface is critical for improving sustainable agriculture [33, 38, 39] and improving models regarding plant’s efficiency to sequester anthropogenic CO_2_ emissions [29, 30].

Bacteria have evolved numerous strategies to overcome environmental Pi scarcity, which are almost exclusively regulated by a two-component system, usually referred to as PhoBR (PhoB, DNA binding response regulator; PhoR, transmembrane histidine kinase) [13]. Several P-scavenging strategies are common to almost all bacteria, the most common being possession of a high affinity Pi transport system, PstSABC, as well as inducing periplasmic and OM-bound phosphatases [13, 15, 40]. Several studies have shown that Bacteria predominantly secrete non-specific alkaline phosphatases (APases) comprising three distinct forms (PhoA, PhoD and PhoX) whilst a few cultured strains secrete acid phosphatases (AcPases, class I, II and III) in response to Pi limitation [13–16, 41]. However, recent evidence from genomic and metagenomic datasets suggest only half possess these characterised P-mobilising enzymes [42].

Here, we isolated *Bacteroidetes* strains from the rhizosphere of the economically important crop *Brassica napus* L. (Oilseed Rape, OSR), grown under field conditions, to determine the Pi-mobilisation potential of this enigmatic phylum. After initially observing atypical constitutive phosphatase activity in all our *Bacteroidetes* isolates we performed a comprehensive multi-layered omics approach to identify the genetic loci responsible for this unique trait. In doing so we also discovered *Flavobacterium* possess an extremely high potential for transforming rhizosphere-associated P_o_ through unique uptake and degradation systems.

## Materials and methods

### Isolation and growth medium used for cultivating *Bacteroidetes*

*Flavobacterium johnsoniae* DSM2064 (UW101) was purchased from the Deutsche Sammlung von Mikroorganismen und Zellkulturen (DSMZ). *Flavobacterium* sp. F52, a bell pepper rhizobacterium [43] and *Flavobacterium* sp. L0A5, a desert plant rhizobacterium (Cytryn, unpublished data) were both kindly donated from the Cytryn Lab, Agricultural Research Organisation, Israel. Strains were routinely maintained on casitone yeast extract medium (CYE) [44]. *Pseudomonas* strains, previously described in [15], were maintained in Luria-Bertani medium. To isolate all other strains a modified R2A medium developed by [45] was used. Cyclohexamide (100 mg L^−1^) and Kanamycin (40 mg L^−1^) were added to reduce fungal and non-Flavobacterial growth, respectively. To investigate the effect of Pi‐depletion on the *Flavobacterium* strains, each was grown (*n* = 3) in a Minimal A medium adapted from [15]. Carbon source, glucose 3.6 g L^−1^ and 20 mM Bis/Tris buffer pH 7.2 was used instead of HEPES and KH_2_PO_4_ added to a final concentration of either 50 μM or 1.4 mM. A final concentration of NH_4_Cl 1 mM, FeCl_2_ 10 mg L^−1^ and KH_2_PO_4_ 1.4 mM was used for the N-limited medium. A final concentration of NH_4_Cl 8.4 mM and KH_2_PO_4_ 1.4 mM with no Fe added was used for the Fe-limited medium. The control contained an excess of all nutrients. Cells were harvested at mid exponential phase (OD_600nm_ 0.4–0.8) after 16–20 h.

### Isolation of *Bacteroidetes*

For isolation, two field trials growing *Brassica napus* L. (OSR) were utilised. The first was located at Warwick Crop Centre (April 2017), University of Warwick, Wellesbourne and the other was located at Sonning Farm, University of Reading (Sept. 2017). Samples were collected during the flowering stage at Warwick Crop Centre and during the four-leaf stage at Sonning Farm. Plants were removed from soil and shaken to remove all loosely adhering soil. Roots containing tightly adhering soil were cut and washed (shaken vigorously) in 10 mL phosphate-buffered saline solution to remove rhizosphere soil. A serial dilution of the resulting soil solution was set up and plated onto either modified R2A or CYE. Individual colonies were picked and sub-cultured prior to identification by 16S rRNA gene analysis.

### Genome sequencing of the *Flavobacterium* isolates

The OSR isolates were grown on CYE and cells were collected from plates by scraping off bacterial colonies. Cells were then sent to the Microbes NG unit (https://microbesng.uk/) at the University of Birmingham and data are deposited in the European Nucleotide Archive database. For assistance, amino acid sequences for the complete CDS profile of all newly isolated strains are attached to the Tables file (S16–S22).

### Quantification of phosphatase activity

The protocol was adapted from Lidbury et al. [15]. Briefly, 0.5 ml cell cultures (*n* = 3) for both Pi-replete and Pi-deplete growth conditions were directly incubated (30°C at 160 rpm) with 4 mM *para*‐nitrophenyl phosphate (*p*NPP) using a 100 mM *p*NPP stock solution in 20 mM Tris HCl, pH 7.2. The pH of the reaction mixture was equivalent to the culture medium and was not altered by addition of *p*NPP. The reaction was stopped using 2 mM NaOH once a colour change was detected. A colour change typically occurred within 5–20 min unless activity was very low (*Pseudomonas* spp.), in which case reactions were stopped after 1 h. All raw absorbance values were lower than A405nm 1. For each strain and growth condition, A405nm measurements were corrected by subtracting A_405nm_ measurements for reactions immediately stopped with NaOH. Normalisation against the culture optical density (OD_600_) was performed and the rate was calculated and expressed h^−1^. A standard curve for *para*‐nitrophenol was generated using a range of known concentrations (0.25, 0.5, 1, 2, 4 mM). A qualitative plate assay using the substrate XP (5-Bromo-4-chloro-3-indolyl phosphate) was also established to confirm phosphatase activity.

### Multi-omics analysis of nutrient-limited *F. johnsoniae*

To capture the transcriptome, RNA was extracted using the RNeasy Mini Kit (QIAGEN) following the manufacturer’s instructions. RNA-seq library preparation, sequencing and standard bioinformatics analysis were performed by Novogene according to the company pipeline. A full description of the method is provided in Supplementary materials. To capture the cellular proteome (CP) and exoproteome (XP) we followed the method described by Lidbury et al. [15]. A full description of the method is provided in Supplementary materials.

### Protein fractionation of the soluble *F. johnsoniae* CP

Cells (500 ml culture) were grown under Pi-deplete conditions and harvested as already described above. Detailed methodology for all three fractionation steps are described in Supplementary materials. An initial fractionation step was performed using size-exclusion chromatography via gel filtration. Fractions were assayed for phosphatase activity as described above. 1D-SDS PAGE analysis revealed the active fractions (peaks 1 and 2) contained a complex mixture of proteins, therefore a second fractionation step was performed on each peak. Both phosphonate-affinity chromatography (peak 1) and anion-exchange (Na gradient) chromatography (peak 2) was performed prior to activity assays and peptide identification.

### Comparative genomics analyses

The online platform **I**ntegrated **M**icrobial **G**enomes & Microbiomes server at the **J**oint **G**enome **I**nstitute (IMG/JGI) was used to perform the majority of comparative genomics analyses described in this study. Genomes were stored in Genome sets and BLAST searches (Min. similarity 30%, *E*-value e^−50^) were set up using the ‘jobs function’.

To determine genome completeness of the OSR isolates, orthologs of core metabolic genes [46] were identified in DSM2064 and F52 using IMG/JGI. Local BLASTP was then performed on the OSR isolates and LAO-5 using DSM2064 orthologs as the query. To generate and annotate ORFs, a local version of the Prokka pipeline [47] was performed. Comparative genomic analyses of the *Flavobacterium* strains used in this study were performed using a local version of BLAST+. In most cases ORFs found in DSM2064 were used as the query, unless this strain lacked a given ORF. A relatively high stringency was set (e^−90^). Any hits with low scores were manually checked to determine whether they were true orthologs or paralogs.

Structural homology analysis of the various uncharacterised Pi-acquisition proteins was performed using the online servers for SWISS-model homologue analysis (https://swissmodel.expasy.org/) and **P**rotein **H**omology/analogy **R**ecognition **E**ngine Version 2.0 (PHYRE2, http://www.sbg.bio.ic.ac.uk/~phyre2/html/page.cgi?id=index). All searches were performed using the default parameters.

Phylogenetic analyses were performed using IQ-Tree [48] using the parameters -m TEST -bb 1000 -alrt 1000. Thus, the most suitable model was chosen by the software. Evolutionary distances were inferred using maximum-likelihood analysis. Relationships were visualised using the online platform the **I**nteractive **T**ree **o**f **L**ife viewer (https://itol.embl.de/).

All statistical analyses and data visualisation were performed using the vegan, ggplot2, ggfortify, tidyr, plyr, serration packages in Rstudio (1.2.5033).

## Results

### Root-associated *Bacteroidetes* display ‘unusual’ constitutive and strong inducible phosphatase activity

**Fig. 1:**
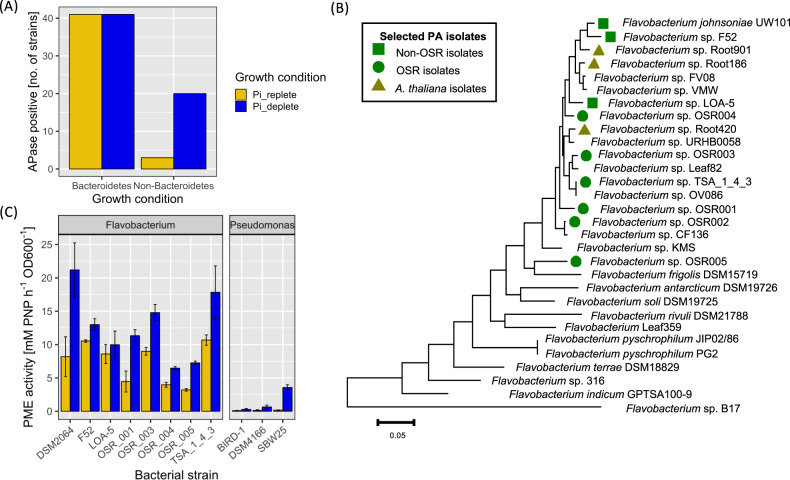
Phosphomonoesterase activity observed in plant-associated *Bacteroidetes*. **A** Phosphatase (monoesterase) activity of *Bacteroidetes* (*n* = 42) and non-*Bacteroidetes* strains (*n* = 20) isolated from the rhizosphere of field-grown Oilseed Rape (OSR) grown on glucose as the carbon source. **B** Phylogenetic analysis of *Flavobacterium* isolates using a multi-locus approach with ten single-copy core genes (as previously described by [18]). Evolutionary distances were inferred using the maximum-likelihood method in MEGA 7. **C** Phosphomonoesterase activity of whole-genome-sequenced OSR strains, non-OSR *Flavobacterium* strains and three *Pseudomonas* rhizosphere strains grown on glucose as the carbon source. Results are the mean of triplicate assays and error bars denote ± standard deviation.

Using a selective medium [43], we isolated numerous *Bacteroidetes* strains from field-grown OSR rhizosphere soil (Two locations; Sonning Farm, Reading, UK and Warwick Crop Centre, Wellesbourne, UK). Most isolated *Bacteroidetes* were related to *Flavobacteriaceae* and to a lesser extent *Sphingobacteriaceae* (Fig. S1). In addition, several kanamycin-resistant non-*Bacteroidetes* strains were still isolated. A random subset of 42 *Bacteroidetes* isolates and 20 non-*Bacteroidetes* (predominantly *Gammaproteobacteria*) were screened for phosphatase (phosphomonoesterase [PME]) activity (Fig. 1A). Strikingly, all *Bacteroidetes* isolates tested exhibited constitutive phosphatase activity i.e. irrespective of Pi availability, in contrast to just 3/20 non-*Bacteroidetes* isolates. All OSR isolates exhibited inducible phosphatase activity when grown under Pi-deplete conditions.

Five OSR rhizosphere strains, representing the breadth of terrestrial *Flavobacterium* diversity (Fig. 1B), were selected for further analysis of their in vitro PME activity. For comparison, the model soil bacterium *Flavobacterium johnsoniae* DSM2064 (hereafter DSM2064) [42], two previously-isolated rhizosphere strains *Flavobacterium* sp. F52 (F52) and *Flavobacterium* sp. L0A5 (LOA5) [41], and three plant-growth promoting *Pseudomonas* spp. strains [15, 49] were also assayed. After overnight growth on glucose as a carbon source, due to the conversion of excess sugar to organic acid, the pH of the medium for all strains dropped to pH 6.1 ± 0.4. Whilst the strength of both the constitutive and inducible phosphatase activity varied, all *Flavobacterium* strains exhibited constitutive activity (Fig. 1C). The *Pseudomonas* isolates only possessed inducible phosphatase activity, consistent with their possession of well-known Pi-sensitive phosphatases [15]. Furthermore, *Flavobacterium* inducible phosphatase activity was far superior to the *Pseudomonas* strains (Fig. 1C).

### Phosphate depletion elicits a major regulatory response in *Flavobacterium johnsoniae*

**Fig. 2:**
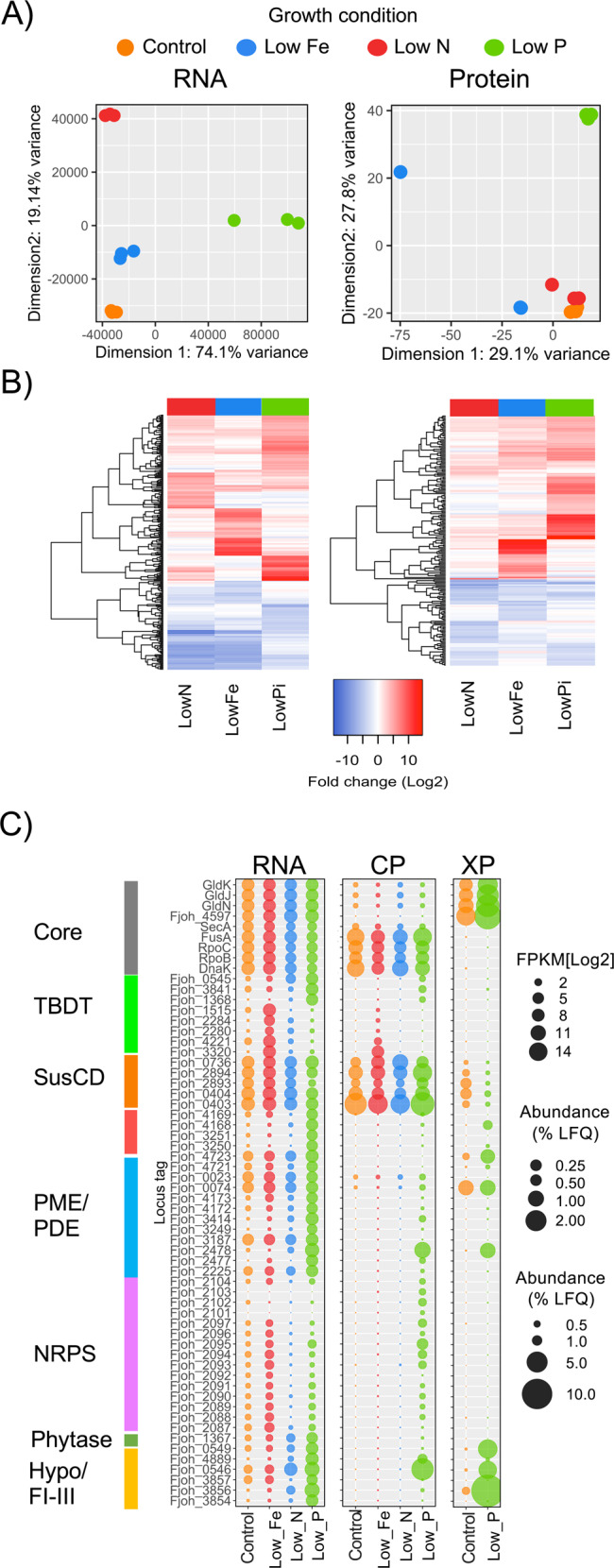
Transcriptomic and proteomic analyses of *F. johnsoniae (*DSM2064) in response to growth under different nutrient stress conditions. **A** Multidimensional scaling (euclidean distance) analysis of RNA and protein content in DSM2064 sampled after 18 h growth. One Fe replicate contained much less protein, hence its separation in the multidimensional scaling. **B** Selected genes and corresponding proteins showing significant (*T* test = *P* < 0.05, adjusted FDR = 0.05) differential expression/abundance from the control growth condition. **C** The relative abundance of selected genes in the transcriptome (% abundance based on FPKM [Log_2_]) and corresponding proteins (% abundance based on raw label-free quantitative values) detected in either the total cellular proteome (CP) or exoproteome (XP). Genes/proteins were categorised into various functional guilds i.e. Core/housekeeping (grey), TonB-dependent transporter (TBDT, green), SusCD-like transport system (red), phosphatase-like enzymes (sky blue), a non-ribosomal secondary metabolite cluster (NRPS, purple), phytase (dark green), hypothetical lipoproteins, FI/II/III (orange). Results in (B) and (C) are the mean of triplicate cultures.

To determine the phosphatases responsible for generating both the inducible and constitutive phosphatase activity, the model bacterium DSM2064 was challenged with Pi-limiting growth conditions and subjected to both transcriptomic and proteomic analyses. The latter comprised analysis of both CP and XP. Iron (Fe) and nitrogen (N)-limiting growth conditions were also established to act as additional controls in addition to the high Pi/Fe/N controls (Fig. 2). DSM2064 exhibited a clear transcriptomic response to all three growth-limiting conditions, but the proteomic response was most pronounced during Pi-depletion (Fig. 2A). Specific genes and gene clusters were differentially expressed under the three distinct nutrient-limiting growth conditions (Fig. 2B, C, Tables S1 and S2). Fe-regulated and N-regulated genes and their proteins are presented in Tables S1 and S2. Pi-depletion resulted in the greatest number of upregulated transcripts (_log2_FC > 4, *Q* value *P* < 0.05; Pi = 95 [avg. FC = 6.54], N = 57 [avg. FC = 4.95], Fe = 47 [avg. FC = 7.69]) and corresponding proteins. This included an unusually high number of genes predicted to encode PMEs and phosphodiesterases (PDEs) (Table 1). Whilst several P-responsive loci have no known function, their tight repression under N and Fe limitation suggests these unique proteins play a primary role in scavenging P. These uncharacterised low-Pi-inducible loci included four hypothetical lipoproteins (Fjoh_0546, Fjoh_0549, Fjoh_3856, Fjoh_4889), two of which were located downstream of low-Pi-inducible TBDTs. Significantly, three of these hypothetical lipoproteins represented a major fraction of the Pi-deplete XP (Table 1 and Fig. 2C). Whilst some OM-transport systems related to TBDTs and SusCD-like complexes were either constitutively expressed or induced in response to Fe-depletion (archetypal function), we identified three TBDTs and two SusCD-like complexes (hereafter termed **P**hosphate **u**tilisation **s**ystem (Pus)), that were induced under Pi-depletion only (Table 1 and Fig. 2C). Interestingly, we also observed the expression of a non-ribosomal peptide cluster in response to Pi-depletion whose expression appeared to be post-transcriptionally regulated (Fig. 2C).

**Table 1:** Expression (transcriptomic and proteomic) of P-acquisition and P-stress response loci in *F. johnsoniae* DSM0264.

						Transcriptome		Whole cell proteome			Exoproteome		
Predicted function	Locus_tag	Gene symbol/description	Genomic context	Predicted localisation	Pfam accession	FC	FDR (*p*-adj)	DE	−Log *p* value	*q* value	DE	−Log *p* value	*q* value
Phosphomonoesterase	Fjoh_0023	PafA	Orphan	Periplasm	pf01663	0.26	5.52E−01	0.82	3.61	0.00130526	-2.72	2.28	0.00
	Fjoh_1367	Phytase	Phytase	OM	pf02333	5.76	2.01E−36	6.83	0.51	9.31E−03	ND	ND	ND
	Fjoh_2478	PhoX_like	PhoX	OM	pf05787	12.50	2.74E−142	11.34	0.28	0.00E+00	8.23	4.22	0.00E+00
	Fjoh_2225	Putative APase	Orphan	Periplasm	pf01676	4.25	7.07E−34	6.20	0.39	0.00E+00	6.63	4.04	0.00E+00
	Fjoh_3187	PhoA2	Orphan	Periplasm	pf00245	1.59	0.28508	3.89	3.66	1.36E-03	0.03	0.01	9.73E-1
	Fjoh_3249	PhoA1 (putative diesterase activity)	PUSII	Periplasm	pf00245, pf13653	7.40	4.39E−61	4.87	0.11	0.00E+00	ND	ND	ND
	Fjoh_3414	Hypothetical Lipoprotein - putative phosphatase	Orphan	OM/IM	IPR011044, IPR011048, IPR015943	10.32	1.41E−90	9.09	0.08	0.00E+00	5.00	2.53	2.71E-03
Phosphodiesterase	Fjoh_0074	Endo/exonuclease/phosphatase	Orphan	OM	NA; IPR011050	1.15	4.17E−02	1.43	2.86	6.64E−03	0.57	0.56	0.23
	Fjoh_3851	Polyphosphate kinase_like/PlcP D	FI cluster/ PUSIII	Cytoplasm	pf02503, pf13089, pf13090	7.80	6.37E−75	ND	ND	ND	ND	ND	ND
	Fjoh_4172	Phosphoinositide phospholipase C, Ca^2+^ -dependent, phosphodiesterases	PUSI	Periplasm	pf00149	6.44	2.87E−56	6.89	0.94	2.11E−03	6.03	1.03	6.15E-02
	Fjoh_4173	3′,5′-cyclic AMP phosphodiesterase CpdA	PUSI	Periplasm	pf16670	7.17	4.47E−69	4.48	0.37	3.79E−03	2.18	2.03	5.44E-03
	Fjoh_4721	Extracellular nuclease, EndA1	Orphan	OM	pf00041,pf00932, pf04231	4.66	3.19E−27	0.71	3.50	2.17E−02	3.70	3.92	0.00E+00
	Fjoh_4723	Extracellular nuclease, EndA2	Orphan	OM	pf00041,pf00932, pf04231	4.13	1.02E−32	ND	ND	ND	2.88	4.34	0.00E+00
Transporter/Binding	Fjoh_0542	OprP	PhoBR operon	OM	pf07396	-1.3	0.24533	ND	ND	ND	ND	ND	ND
	Fjoh_0545	Tdbt	PhoBR operon	OM	pf07715, pf13715, pf14905	8.02	8.21E−84	6.52	0.49	7.91E-03	5.38	5.68	0.00E+00
	Fjoh_0546	Hypothetical Lipoprotein - FII	PhoBR operon	OM	NA; IPR011050	9.91	3.67E−114	11.19	0.16	6.13E−03	6.47	4.57	0.00E+00
	Fjoh_0549	Hypothetical Lipoprotein	PhoBR operon	OM	pf07705	7.59	1.67E−71	5.06	1.72	3.57E−03	7.05	3.64	0.00E+00
	Fjoh_1368	Tbdt	Phytase	OM	pf07715; pf13715	9.22	1.84E−30	8.94	0.65	0.00E+00	3.72	2.41	5.29E-03
	Fjoh_3250	PusD2	PUSII	OM	pf07980, pf14322	8.94	1.49E−79	5.47	3.11	0.00E+00	8.85	4.43	0.00E+00
	Fjoh_3251	PusC2	PUSII	OM	pf00593, pf07715, pf13715, pf16344	9.03	7.60E−87	4.53	0.24	0.00E+00	ND	ND	ND
	Fjoh_3841	Tbdt	Orphan	OM	pf13620	7.69	2.64E−81	8.44	0.11	0.00E+00	8.34	4.60	0.00E+00
	Fjoh_3856	Hypothetical Lipoprotein - FI	FI cluster/ PUSIII	OM	NA, lipoprotein	14.47	5.36E−31	6.33	0.01	1.48E−3	5.39	3.07	0.00E+00
	Fjoh_3857	Tbdt	FI cluster/ PUSIII	OM	pf07715, pf13715	12.50	7.00E−100	5.18	4.08	1.41E-03	3.73	2.48	3.38E-03
	Fjoh_4168	PusD1	PUSI	OM	pf07980, pf14322	10.17	7.54E−94	6.23	1.84	0.00E+00	8.55	4.99	0.00E+00
	Fjoh_4169	PusC1	PUSI	OM	pf00593, pf07715, pf13715	10.66	8.26E−101	3.17	0.01	1.33E−01	ND	ND	ND
	Fjoh_4889	Hypothetical Lipoprotein - FIII	Orphan	OM	NA	11.56	4.44E−118	9.13	1.42	0.00E+00	9.56	4.13	0.00E+00
Lipid remodelling	Fjoh_0541	Glycosyl transferase	PhoBR operon	Cytoplasm	pf04101	5.42	2.94E−49	7.04	5.08	0.00E+00	ND	ND	ND
	Fjoh_4577	PlcP	Orphan	Cytoplasm	pf12850	8.59	2.12E−77	ND	ND	ND	ND	ND	ND
Accessory	Fjoh_0543	PhoR	PhoBR operon	Cytoplasm	pf00512, pf02518	0.76	0.056401	ND	ND	ND	ND	ND	ND
	Fjoh_0544	PhoB	PhoBR operon	Cytoplasm	pf00072, pf00486	3.06	9.42E−20	3.23	4.49	0.00E+00	ND	ND	ND
	Fjoh_2477	MauG_like	PhoX	Cytoplasm	pf03150	9.58	3.54E−95	7.33	0.64	8.36E−03	ND	ND	ND
	Fjoh_3252	FecR_like regulator	PUSII	Cytoplasm	pf04773, pf16344	9.88	7.70E−61	ND	ND	ND	ND	ND	ND
	Fjoh_3853	C-terminal peptidase S41	FI cluster/ PUSIII	Periplasm	pf03572	9.08	9.92E−74	ND	ND	ND	4.44	2.79	0.00E+00
	Fjoh_3854	T9SS-secretion domain, lipoprotein	FI cluster/ PUSIII	OM	NA,	9.64	1.90E−66	3.30	0.65	6.04E−03	5.75	4.29	0.00E+00
	Fjoh_3855	T9SS-secretion domain,	FI cluster/ PUSIII	OM	NA	10.04	7.54E−94	ND	ND	ND	8.61	3.90	0.00E+00
	Fjoh_3858	FecR_like regulator	FI cluster/ PUSIII	Cytoplasm	pf04773, pf16344	9.79	9.56E−90	1.53	1.75	7.02E−02	ND	ND	ND
	Fjoh_3859	Sigma factor - regulator	FI cluster/ PUSIII	Cytoplasm	pf04542, pf08281	7.94	2.29E−70	ND	ND	ND	ND	ND	ND
	Fjoh_4171	ECF Sigma factor	PUSI	Cytoplasm	pf04542, pf08281	8.88	1.19E−70	0.05	3.82	9.50E−01	ND	ND	ND

Predicted function is based on in silico sequence analysis. Where applicable, supporting pfam or INTERPRO domains are provided. Genomic context is given in terms of the genetic neighbourhood each gene was identified. Predicted localisation is based on the presence of a leader sequence and/or lipobox. ‘Orphan’ denotes genes not associated with any other predicted P-acquisition genes.

*OM* outer membrane, *IM* inner membrane, *PUS* phosphate utilisation system, *DE* differential expression, *ND* not detected, *FC* fold change.

### *F. johnsoniae* DSM2064 expresses a high number of phosphatases

Further in silico analyses of the putative PMEs and PDEs revealed DSM2064 has a very high potential to mineralise P_o_ (Table 1). Some were low-Pi-inducible whilst others were constitutively expressed, suggesting a constant reliance on P-acquisition from Po. We previously demonstrated that PME activity in *Pseudomonas* spp. was produced by non-cytoplasmic PMEs, indicating *p*NPP does not cross the cytoplasmic membrane [15, 50]. Most PMEs and PDEs were predicted to possess N-terminal signal peptide sequences (periplasmic) and a subset of these also possessed predicted lipobox sequences (OM-anchored). Four putative PMEs likely contributed to the observable PME activity in DSM2064. Sequence and structural homology searches revealed two predicted periplasmic PMEs (Fjoh_3187, Fjoh_3249) were related to PhoA [51] (Fig. S2) and one predicted OM-anchored PME (Fjoh_2478) was distantly related to PhoX [16] (Fig. S3). Fjoh_3249 (PhoA1) Fjoh_3187 (PhoA2) had varied expression profiles and neither were abundant in the CP or XP (Table 1 and Fig. 2C). Fjoh_2478 was heavily transcribed and represented a major component of the XP during Pi-depletion (Table 1 and Fig. 2C). 9/10 key residues found in the PhoX active site were conserved in Fjoh_2478 (Fig. S3). However, phylogenetic analysis revealed *Flavobacterium* PhoX is distinct from the previously defined PhoXI or PhoXII clades [16] (Fig. S4). Indeed, the predicted active site architecture for DSM2064 PhoX and surface electrostatic potential are quite different to previously characterised PhoX (Fig. S3), suggesting a potential difference in enzyme kinetics, pH range and substrate preference [52]. Similar to *Pseudomonas* spp. OM-anchored PMEs [45], we hypothesised Fjoh_2478 contributed most of the inducible PME activity in DSM2064. The fourth putative PME (periplasmic), Fjoh_0023, was expressed in all growth conditions, irrespective of Pi availability (Fig. 2C and Table 1). This predicted periplasmic phosphatase has high sequence (44% identity, e = 5^e−159^) and structural homology (Fig. S5) to the Pi-irrepressible phosphatase PafA, identified in the human opportunistic pathogen *Elizabethkingia* (formerly *Chryseobacterium*) *meningosepticum* [53].

PhoA, PafA and PhoX all mineralise *p*NPP in vitro [16, 40, 50, 53]. Therefore, to determine if these four putative PMEs contributed to PME activity in DSM2064, the soluble CP, extracted from cells grown under Pi-depletion, was fractionated prior to screening for PME activity. Two major peaks of PME activity were detected across the fractionated CP and both of these were subjected to a further fractionation step (See Supplementary methods and Fig. S6). For peak 1, Fjoh_0023 was the major protein whose abundance was positively correlated with PME activity (*P* < 0.01, Fig. S6, Table S4). For peak 2, PhoA2 (Fjoh_3187) was the second most abundant protein detected in the active fraction, whilst another inducible periplasmic APase Fjoh_2225 (Table 1) was also detected, albeit at a lower abundance (Fig. S6 and Table S5).

### *F. johnsoniae* expresses distinct polysaccharide utilisation loci-type gene clusters in response to Pi-depletion

**Fig. 3:**
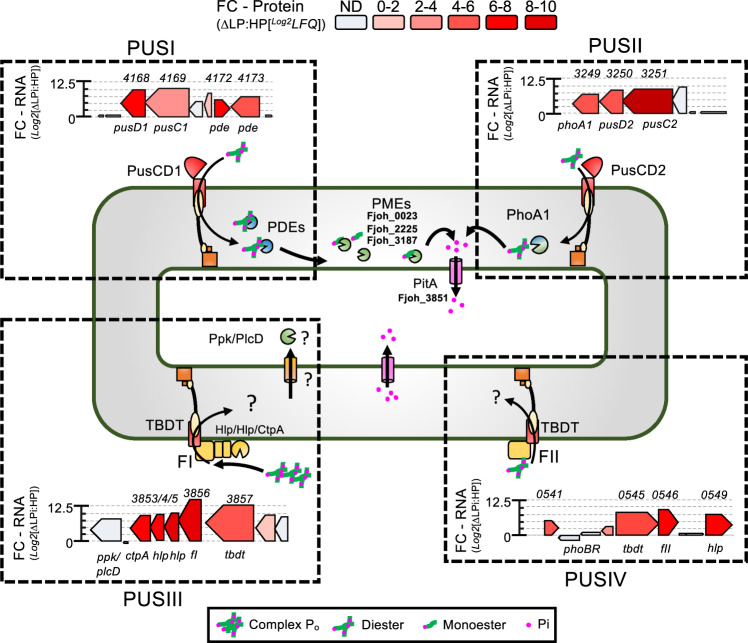
Expression of putative Pi-sensitive polysaccharide utilisation loci in *F. johnsoniae*. Intergration of transcriptomic (*y* axis on genetic neighbourhood maps) and proteomic data (colour gradient on genetic neighbourhood maps) with in silico function prediction/cellular location used to generate a proposed model for the newly identified Phosphate utilisation systems in *Flavobacterium johnsoniae* DSM2064. Locus tags for selected ORFs are given, omitting ‘fjoh_’. Results shown are the mean of triplicate cultures in each growth condition. Abbreviations: FC fold change, LFQ label-free quantification, PusCD phosphate utilisation system transporter complex – SusC forms the transmembrane pore and SusD is the cell-surface ligand-binding protein, FI/FII, hypothetical lipoproteins with homology to polygalacturanase, Ppk/PlcD polyphosphate kinase/phosphatidylinositol phospholipase-like, PME phosphomonoesterase, PDE phosphodiesterase, PitA high velocity inner membrane Pi symporter; *ctpA* – S41 peptidase.

*Bacteroidetes* are best-known for their possession of numerous PULs, typified by the presence of *susCD-like* complexes, enabling them to efficiently acquire and degrade complex polymeric carbon molecules [8, 19]. We identified four gene clusters, heavily induced during Pi-depletion, that are predicted to share similar mechanisms with traditional PULs for OM-binding, OM-transport, and periplasmic degradation of organic substrates (Fig. 3). The first two, labelled PUSI and PUSII, possessed the two low-Pi-inducible PusCD complexes, PusCD1 (Fjoh_4168/9) and PusCD2 (Fjoh_3250/1) (Fig. 3). Unlike traditional PULs that contain hydrolytic enzymes found in the CAZy database [22, 54], PUSI and PUSII were co-localised with two of the differentially expressed periplasmic PDEs (PUSI, Fjoh_4172; Fjoh_4173), or the PME, PhoA1 (PUSII, Fjoh_3249). PhoA1 is predicted to possess an additional domain (pf13653), unique for PhoA, potentially extending its substrate range to target specific phosphodiesters (Table 1). In agreement, PhoA1 is predicted to have a more accessible active site, a prerequisite for catalysis of phosphodiesters [55] (Fig. S2).

Two more PUS gene clusters (here labelled PUSIII and PUSIV) were also identified. PUSIII contained a hypothetical lipoprotein, hereafter termed F(form)I, encoded by *fjoh_3856*, that was the most differentially transcribed gene and the most abundant exoprotein (18.7%) suggesting an important role in combatting Pi-depletion (Table 1 and Fig. 2C). The remaining ORFs in PUSIII encoded a TBDT/SusC-like porin, various OM hypothetical proteins, an OM protease and a putative cytoplasmic PDE, all of which were induced in response to Pi-depletion (Fig. 3). In PUSIV, the ORF encoding another related hypothetical lipoprotein, hereafter termed F(form)II, is co-localised in a Pi-sensitive gene cluster containing *phoBR* and ORFs encoding other heavily expressed proteins (Fig. 3). FI, FII and a third related orphan lipoprotein, termed FIII, all appear to be related to each other (Fig. S7) and share modest sequence homology (31–36% identity, 50–54% coverage) with a low-Pi-inducible lipoprotein of *Caulobacter crescentus* that has an accessory role in producing phosphatase activity [56]. Furthermore, structural homology modelling revealed these proteins may have a role in binding or hydrolysing plant glycans or other complex plant-cell wall molecules, similar to that of the *Aspergillus niger* polygalacturanase [57] (Fig. S7). Combining all these data, it is clear that DSM2064 invests a large amount of cellular resources into expressing several PUS gene clusters that we predict target P_o_ molecules.

### The unique *Flavobacterium* PHO regulon is largely conserved across plant-associated isolates

**Fig. 4:**
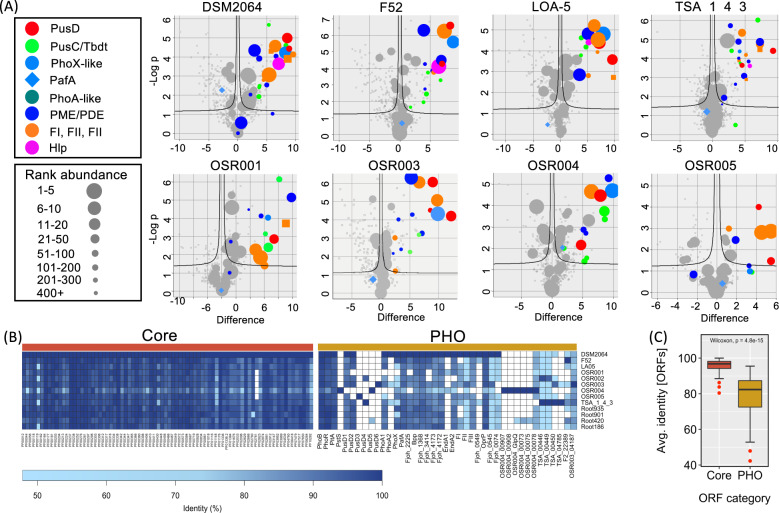
Identification of P-acquisition proteins and their corresponding loci in several plant-associated *Flavobacterium* strains. **A** Volcano plots of exoproteome data illustrating both the difference in the log_2_ label-free quantitative values (LFQ) intensity between Pi-replete and Pi-deplete treatments and the log probability of the observed difference. Dashed lines represent the threshold for significant *P* values adjusted using FDR = 0.03 (*Q* values). The rank abundance of each protein is denoted by dot size. The top 30 proteins detected in the Pi-deplete exoproteomes are highlighted. In addition, the rank abundance of predicted P-acquisition proteins is also given. Data points represent the means of triplicate cultures sampled from each growth condition. Abbreviations: PusD phosphate utilisation system-like outer membrane binding domain, PhoX alkaline phosphatase PhoX-like, PusC/Tbdt including TonB-dependent transporter proteins, APases putative phosphatases, FI FII, FIII, hypothetical exoproteins that share weak homology with pectate lyases/polygalacturonases, PafA Pi-irrepressible phosphatase, Hlp hypothetical lipoprotein located in phoBR operon. **B** Occurrence and similarity (% identity) of ORFs related to core/housekeeping processes (maroon) and P-acquisition (gold) amongst the *Flavobacterium* strains. **C** Box–whisker plots illustrating the mean identity of core and P-acquisition (PHO) ORFs. Red dots denote outliers.

To better determine how important these unique Pi-acquisition loci are for root-associated *Flavobacterium*, we performed whole-genome sequencing and exoproteomics on the OSR rhizosphere strains previously tested for their phosphatase activity (Fig. 1B, C). General genomic characteristics for the newly isolated *Flavobacterium* strains are presented in Table S6. A comprehensive analysis of all the XP datasets are presented in Tables S7–S13. PafA was detected in the Pi-replete and Pi-deplete XPs of all seven (2 non-OSR and 5 OSR) *Flavobacterium* strains (Blue diamonds, Fig. 4A). Conservation in their response to Pi-depletion was also observed (Fig. 4A). This included the abundant expression of proteins associated with the PUSI/II/III/IV gene clusters, the distinct PhoX and other PME and PDEs (Fig. 4A). However, all *Flavobacterium* strains displayed microheterogeneity in their response to Pi-depletion. Most notably, this included the expression of phylogenetically distinct PusCD complexes, located within distinct PUS gene clusters (described in detail below) as well as the expression of strain-specific gene clusters.

This fine-scale heterogeneity in their response to Pi-depletion was also apparent in their genomic make up (Fig. 4B). In comparison to ORFs corresponding to core metabolic and housekeeping genes (~80 single-copy representatives), ORFs corresponding to low-Pi-inducible genes had fewer occurrences and greater sequence divergence across the strains (Fig. 4B). The average sequence identity of PHO regulon ORFs (77.9%) was significantly lower (Wilcox test, *P* < 0.001) than the average sequence identity of the core ORFs (95.9%) (Fig. 4C).

### Highly expressed PHO regulon genes occur more frequently in plant-associated *Bacteroidetes* genomes

**Fig. 5:**
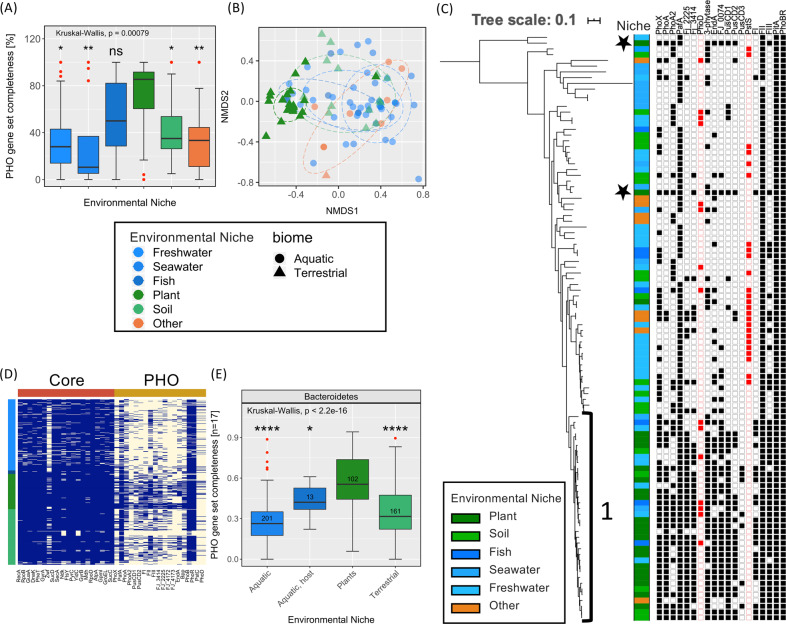
Occurrence of P-acquisition (PHO) genes in the genomes of plant-associated and non-plant-associated *Bacteroidetes* strains. **A** Occurrence of all PHO genes among the 102 *Flavobacterium* strains. Red dots denote outliers in the data and asterisks denote significance levels (Kruskal Wallis; *<0.05, **<0.01, ***<0.001) for differences of occurrence in each group relative to the Plant-associated group. **B** Non-metric multidimensional scaling (Bray-Curtis distances) of P-acquisition gene content among the 102 *Flavobacterium* strains. Ellipses represent euclidean distances from the centre of groups (level = 0.7). **C** The multi-loci maximum-likelihood consensus tree was inferred from a simultaneous comparison of 10 housekeeping and core genes present in 102 *Flavobacterium* isolates. Tree topology and branch lengths were calculated by maximum likelihood using the VT + F + R5 model of evolution for amino acid sequences based on 1022 sites in lQ-TREE software [48]. The presence (filled symbol) or absence (hollow symbol) of selected P-acquisition genes is displayed. PstS and PhoD are highlighted in red. Subclade 1 is denoted by the numeric. Stars represent phylogenetically distinct plant-associated strains. **D** Occurrence of core and PHO genes among 468 *Bacteroidetes* strains isolated from the five environmental niches. **E** Occurrence of all PHO genes, excluding PitA and PhoBR, among the 468 *Bacteroidetes* strains. Red dots denote outliers in the data and asterisks denote significance levels (Kruskal Wallis; *<0.05, **<0.01, ***<0.001) for differences of occurrence in each group relative to the Plant-associated group.

We further investigated the occurrence and diversity of these P-acquisition genes across the *Flavobacteriaceae* family. One hundred two genomes (predominantly from the genus *Flavobacterium*) previously deposited in the IMG/JGI database, representing strains isolated from different environmental niches (using ‘Ecosystem Type’; ‘Study name’; ‘Habitat’) were selected and analysed (Table S14). This included plant-associated, soil (terrestrial), marine (aquatic, sediment), freshwater (aquatic, sediment), fish (marine host-associated) and various ‘other’ microhabitats (wastewater treatment, etc.). The number of newly identified P-acquisition genes occurring in the plant-associated genomes was significantly greater (Wilcox test, *P* = 0.00079) than the number occurring in non-plant-associated genomes (Fig. 5A). This equated to greater similarity in the P-scavenging repertoire between plant-associated strains compared to non-plant-associated strains (Fig. 5B). Almost all plant-associated strains were found within a single subclade (subclade 1, Fig. 5C) demonstrating phylogeny contributed towards the occurrence of the newly identified Pi-acquisition genes within their genomes (Fig. 5C). However, niche adaptation was clearly a strong underlying driver as two plant-associated strains, *Flavobacterium* sp. B17 and *Flavobacterium chilense* DSM24724, that were distantly related to subclade 1, still possessed a high number of P-acquisition genes (black stars, Fig. 5C). Interestingly, ORFs corresponding to the constitutive APase PafA, were found in 90/102 strains suggesting that constitutive phosphatase expression is not confined to plant-associated strains only. The occurrence of the well-known APase *phoD* (15/102) was mostly associated with aquatic strains (13/15). *pstS* (26/102) was absent from subclade 1 and only found in one plant-associated strain (OSR_004) and this was coincident with a lack of *pusCD1*, and to a lesser extent *pusCD2* (Table S14).

Given the niche-associated diversity of PHO regulon genes within the *Flavobacterium* genus, we expanded our comparative genomics analysis across the entire *Bacteroidetes* phylum, still focusing on isolates retrieved from either plant, soil (terrestrial), freshwater or seawater (aquatic), or host-associated strains of aquatic organisms, such as algae or fish (aquatic, host) (*n* = 468, Table S15). Anaerobic isolates inhabiting animal and human gut microbiomes were not scrutinised. The greater heterogeneity in the occurrence of PHO genes versus the core genes was maintained at the phylum level (Fig. 5D), which again resulted in a significantly greater (*P* < 2.2^e−16^) number of PHO genes occurring in plant-associated genomes compared to all non-plant-associated genomes (Fig. 5E). Finally, whilst the average occurrence of PHO regulon genes across the phylum was 35.2 ± 25%, the occurrence of genes encoding the constitutive APase PafA was much greater (78.7%). This suggests, the ubiquitous PafA- dependent constitutive phosphatase expression observed in plant-associated strains here, is a common trait in this phylum.

### Plant-associated *Flavobacterium* have evolved a distinct set of SusCD-like complexes to facilitate Pi-scavenging

**Fig. 6:**
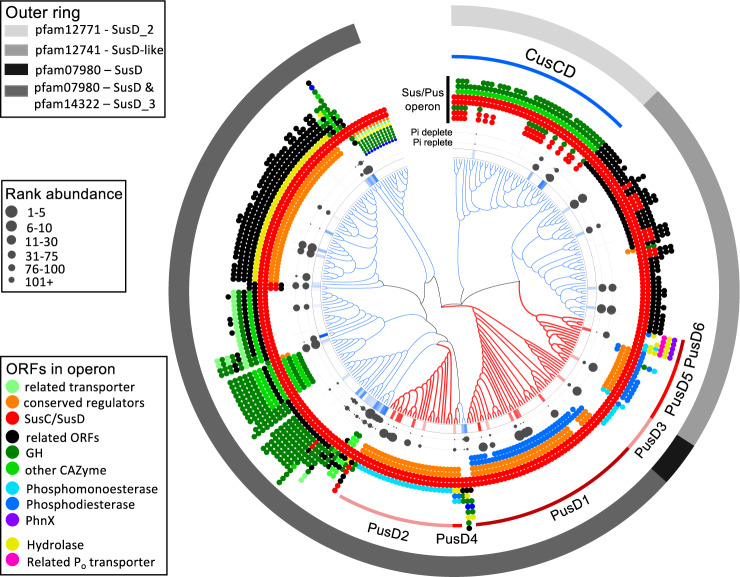
Phylogenetic, expression and genomic relationship analyses of SusD-like homologues in *Flavobacterium*. The maximum-likelihood consensus tree was inferred from a simultaneous comparison of 310 protein sequences related to the SusD lipoprotein. Tree topology and branch lengths were calculated by maximum likelihood using the VT + F + R5 model of evolution for amino acid sequences based on 1022 sites in lQ-TREE software [48]. Clades containing low-Pi responsive PusD proteins are coloured red whilst clades containing Pi-insensitive SusD-like proteins are coloured blue. The first ring expresses the difference in abundance (Pi-deplete: Pi-replete) for all detected SusD-like domains. The second (Pi-replete) and third (Pi-deplete) rings represent the rank abundance against all proteins for detected SusD-like proteins in the various exoproteomes. The genetic neighbourhoods for each SusC/D-like complex are illustrated by the coloured dot plots. Numericals 1–6 denotes the six PusD forms identified in this study. CusCD denotes the experimentally characterised chitin utilisation system [25]. The outer ring defines the pfam domains associated with each SusD form (predicted by IMG/JGI).

In total, we detected the expression of six distinct PusCD complexes across the eight *Flavobacterium* strains (Fig. 4A). All strains also expressed several Pi-insensitive SusCD-like complexes, in agreement with their predicted specialisation in plant glycan degradation [18] (Table S3). To compare the phylogeny of these Pi-sensitive and Pi-insensitive SusCD-like complexes, the SusD-like domains from all complexes detected in the *Flavobacterium* XPs were aligned (Fig. 6). In addition, the corresponding PusD/SusD-like homologues from the 102 *Flavobacterium* genomes were also included. The various PusD and SusD-like forms made up three distinct subclades evidenced by their different pfam domains (Fig. 6, outer ring). PusD was polyphyletic among various SusD-like subclades indicating multiple evolutionary events occurred during the generation of the various PusCD complexes. However, the predicted gene clusters containing the six PusCD forms were clearly distinct from the Pi-insensitive SusCD-like gene clusters. SusCD-like complexes were co-localised predominantly with carbohydrate processing enzymes, whilst all six PusCD complexes, as already described for PUSI and PUSII in DSM2064, were co-localised with PMEs, PDEs, as well as phosphonate-degrading enzymes and inner membrane P_o_ transporters (Fig. 6). All PusCD-containing PUS gene clusters differed, suggesting a distinct role in P_o_-acquisition for each. PUSI, containing PusCD1, and the two predicted periplasmic PDEs, was the most common PUS system found in *Flavobacterium* revealing these loci likely play an important role in scavenging ubiquitous phosphodiesters (most likely lipid headgroups and nucleic acids) found in the soil/rhizosphere.

## Discussion

Competition for P in the rhizosphere is incredibly intense [30, 34]. Hence, understanding how *Bacteroidetes* competes for this scarce nutrient provides valuable insights into the underlying molecular mechanisms responsible for this phylum being a dominant member of the plant microbiome [1–3, 8]. This study provides strong evidence that plant-associated *Bacteroidetes* possess an incredibly high potential for transforming P_o_ to bioavailable Pi. This includes the apparent identification of novel molecular mechanisms for P uptake linked to TonB-dependent transport [58] that differ from previously known bacterial P transport mechanisms [13].

The constitutive PME expression displayed by all *Bacteroidetes* OSR isolates irrespective of Pi concentrations (Fig. 1) is a unique and potentially important trait in the context of improving plant phosphorus uptake at the root–soil interface [33, 39, 59]. Whilst inducible PME activity is high when *Pseudomonas* spp. are grown on succinate (growth medium pH 7–8) [15, 50], a lower pH range (pH 5–7) in the growth medium used here drastically inhibited their PME activity. Thus, *Flavobacterium* spp. may outperform traditional alkaline phosphatase-harbouring rhizobacteria, including *Pseudomonas* spp. [13–16], with respect to P-mobilisation, in an agricultural setting where rhizosphere pH is typically pH 5.5–7.5 [32]. This hypothesis warrants further investigation through protein purification and *in planta* experiments.

Plant-associated *Flavobacterium* expressed a far greater number of PMEs and PDEs than other well-known rhizobacteria [13–16] and not all were silenced by high concentrations of Pi (Table 1). Importantly, the majority of these P-acquisition loci appear to be niche-adapted traits that would allow plant-associated *Bacteroidetes* to overcome competition for P in the rhizosphere, where labile Pi is depleted and replaced by immobilised P_o_ [32, 37]. The *Flavobacterium* PhoX is a prime example of this niche adaptation to the plant microbiome. This phosphatase was one of the major proteins secreted by all plant-associated *Flavobacterium* strains in response to Pi-depletion (Fig. 4) but is only a common genetic feature in the genomes of plant-associated *Bacteroidetes* strains. PhoX is a lipoprotein [60], hence we did not detect this in our soluble fractions (Fig. S6). However, given its predicted localisation in the OM and structural similarity to other characterised PhoX homologues that are responsible for PME activity in other rhizobacteria [16, 47, 60, 61], we predict this enzyme is responsible for most of the elevated inducible PME activity observed in our *Flavobacterium* strains. *Flavobacterium* PhoX is both phylogenetically and structurally distinct from previously characterised PhoX and can be further subdivided into three subclades (Fig. S4). Each subclade is predicted to possess a different active site architecture and surface potential compared to the characterised PhoX [61] (Fig. S3), which may explain why *Flavobacterium* inducible activity is more resilient to lower pH conditions compared to *Pseudomonas* [15, 50].

In contrast, the widespread occurrence of PafA in *Bacteroidetes* isolated from all environmental niches reveals that the unusual constitutive phosphatase expression is almost certainly a common phenotypic trait in this phylum. PafA, whose expression was constant in all eight strains, is a Pi-irrepressible phosphatase that is a unique member of the alkaline phosphatase superfamily whose pH optimum is neutral to slightly acidic and also possesses diesterase activity [53, 55]. Using DSM2064 as the model, PafA was the major protein detected in one of the phosphatase-active protein fractions (Fig. S6), strongly suggesting this enzyme contributes towards the unique constitutive and inducible PME activity in our *Flavobacterium* isolates.

Why *Bacteroidetes* have such a huge diversity of TBDTs and SusCD-like complexes is a recurrent question [5, 22, 58]. Here, we present evidence that suggests novel OM transporters related to those traditionally associated with siderophore and oligosaccharide transport [20–25, 55, 62] may be involved in transporting P_o_ species. All plant-associated *Bacteroidetes* lack common organic phosphorus transporters and the high affinity PstSABC transporter for Pi [13, 15], but instead possess novel low-Pi-inducible PusCD complexes and orphan TBDTs, indicating *Bacteroidetes* favour an alternative mechanism for competitively acquiring P. Given, the pore size of transmembrane TBDTs and SusC-like domains and general role as polysaccharide transporters [19, 58], their co-localisation with ORFs encoding co-expressed predicted periplasmic PME and PDEs (Fig. 6) strongly suggests these transporters have evolved to target P_o_. The co-localisation of a low-Pi-inducible TBDTs with 3-phytase (Fjoh_1368), a well-known P_o_-degrading enzyme [63] supports this hypothesis. Further support is provided by the abundant expression of the uncharacterised FI and FII, located in PUSIII and PUSIV, that both share sequence homology with an uncharacterised PHO regulon lipoprotein in *C. crescentus* [56] as well as structural homology with plant-cell wall degradation enzymes [57]. Thus, when facing Pi-depletion, *Flavobacterium* may target the large reservoir of P_o_ associated with plant root cell walls [64] and the consequence of this unique mechanism warrants further investigation.

Marine and Gut *Bacteroidetes* predominantly acquire carbohydrate substrates through a ‘selfish uptake’ mechanism using SusCD-like harbouring PULs [24, 65]. This selfish mechanism for degrading more recalcitrant organic carbon does not liberate low molecular weight carbon for scavengers in the way the ‘sharing’ mechanisms (extracellular enzyme secretion) can [65]. We have recently demonstrated ‘sharing’ of more recalcitrant P_o_ substrates occurs in *Pseudomonas* spp. through the production of periplasmic and extracellular phosphatases [50]. Based on the data presented here, *Flavobacterium* may be capable of both a sharing and selfish mechanism of P_o_-utilisation that is partially dependent on external Pi concentrations, which may have divergent outcomes for rhizosphere P cycling.

## Conclusions

P_o_ often represents a major fraction of the total P content in both agricultural and non-agricultural soils [59, 66]. This is set to rise with the increased use of organic P fertilisers primarily from manure and other waste materials [31, 59]. Our finding that abundant resident microorganisms of many cropping systems potentially possess novel mechanisms for liberating Pi from P_o_ may influence sustainable approaches to crop production and combat the global phosphorus crisis [24, 29, 34, 59]. Whether these novel and unique molecular mechanisms discovered in *Bacteroidetes* result in an enhanced or broader capability to degrade rhizosphere-associated P_o_ is still an open question, but one which has important consequences for sustainable agriculture. Not only for enhancing phosphorus acquisition in crops, but also developing ‘designer rhizospheres’ through selection, using plant genotyping and environmental conditioning, to enhance plant functionality, such as plant immunity or bioremediation [67]. This study paves the way for further exciting research on the P physiology of *Flavobacterium* strains and their potential exploitation as microbial tools in sustainable agriculture.

## Supplementary information

Supplementary material

Supplementary tables

## Data Availability

The proteomics datasets supporting the conclusions of this article are available in the ProteomeXchange Consortium via the PRoteomics IDEntifications (PRIDE) partner repository with the dataset identifier PXD014380 and 10.6019/PXD014380. The transcriptomics dataset supporting the conclusions of this article are available in the NCBI short read archive (SRA) repository with the dataset identifier Bioproject accession PRJNA635152.
