# Supplementary material for: Niche-adaptation in plant-associated Bacteroidetes favours specialisation in organic phosphorus mineralisation
Source: ISME J. 2020 Nov 30;15(4):1040–55. doi: 10.1038/s41396-020-00829-2 (PMC8115612; doi:10.1038/s41396-020-00829-2)
Supplement: Supplementary file 1 — Supplementary material [file 41396_2020_829_MOESM1_ESM.docx]

**Supplementary material**

**Niche-adaptation in plant-associated Bacteroidetes favours specialisation in organic phosphorus mineralisation**

Ian D.E.A. Lidbury^1,2*+^, Chiara Borsetto^1+^, Andrew R. J. Murphy^1^, Andrew Bottrill^1^, Alex Jones^1^, Gary D. Bending^1^, John P. Hammond^2^, Yin Chen^1^, Elizabeth M. H. Wellington^1^, David J. Scanlan^1^

^1^ School of Life Sciences, University of Warwick, Gibbet Hill Road, Coventry, UK

**^2^** Department of Animal and Plant Sciences, University of Sheffield, Sheffield, UK

^3^ School of Agriculture, Policy, and Development, University of Reading, Earley Gate, Whiteknights, Reading, UK

^+^Both are lead authors

***Corresponding author:** [**I.lidbury@sheffield.ac.uk**](mailto:I.lidbury@sheffield.ac.uk)

**Supplementary Methods**

**Transcriptomic analysis of *F. johnsoniae***

RNA was extracted using the RNeasy Mini Kit (QIAGEN) following the manufacturer’s instructions. Briefly, equal amounts of cells (1-2 mL culture) according to OD_600nm_ were harvested after 16-20 h of growth for all the conditions tested (Controls, Low Pi, Low NH_4_, Low-Fe). RNAprotect^®^ Bacteria Reagent (QIAGEN) was added to the cells as per the manufacturer’s instructions prior to centrifugation and flash-freezing to store the stabilised bacterial pellet at -80°C. After thawing the pellet, an enzymatic lysis and a proteinase K digestion step was performed and RNA was recovered using the RNA binding column provided in the kit. RNA concentration was measured using a Nanodrop (ThermoScientific) and genomic contamination was checked by agarose gel electrophoresis prior to DNase treatment when required. Removal of genomic DNA was performed with TURBO DNase (Invitrogen) as per the manufacturer’s instructions. All RNA samples were run on the 2100 Bioanalyzer (Agilent) to obtain RNA Integrity Number (RIN) and final concentration. RNA-seq library preparations, sequencing and standard bioinformatics analysis were performed by Novogene according to the company pipeline. For each RNA sample provided, a 250-300 bp insert strand specific library with rRNA removal (Ribo-ZeroTM Magnetic Kit) was prepared and 1 GB of raw sequence data obtained. (Bioproject accession PRJNA635152). For review purposes, the following link can be used to access the data (<https://dataview.ncbi.nlm.nih.gov/object/PRJNA635152?reviewer=9nav0b3m2iboav1fiqfkhsvgk4>). Differential gene expression results were independently verified using the DEGUST online platform (<http://degust.erc.monash.edu/>).

**Proteomic analysis of *F. johnsoniae***

For each condition tested, three biological replicates were extracted and analysed. For each sample, 25 mL culture was harvested by centrifugation at 10000 g for 15 min at 4°C. Pellets were recovered, flash-frozen and stored at -80°C until further processing. For the whole cell protein extraction, each cell pellet was thawed on ice and resuspended in 2-2.5 mL of 20 mM Tris-HCl pH 7.8. Cells were lysed using a French press (3 cycles/ samples at 1000 psi) and kept on ice during the whole process. For each lysate 1 mL was centrifuged at 14000 rpm 4°C for 15 min and 0.5 mL of the supernatant was recovered and used for protein quantification using the Bradford reagent (Alfa Aesar) as per the manufacturer’s instructions. 10 μg protein for each sample were mixed with dithiothreitol (DTT) and RunBlue lithium dodecyl sulphate (LDS) loading buffer (Expedeon), heated at 80°C for 10 min and loaded for SDS-polyacrylamide gel electrophoresis (SDS-PAGE) in a precast RunBlue SDS gel 4-20 % (Expedeon). SDS-PAGE was performed with RunBlue SDS Running Buffer (TEO-Tricine) 1X (Expedeon) at 140 V for either 40 min or 10 min. Gels were stained with Instant Blue (Expedeon). The long run gels were visually checked for consistency between replicates while the short run gels were used for the proteomic run.

For the exoproteomes, cells were pelleted at 4,000 rpm 4^o^C in a Beckman benchtop centrifuge prior to gentle hand filtration through 0.45 μm and 0.22 μm pore size PDVF membranes. The remaining supernatant was frozen at -20^o^C until further analysis. Culture supernatants were thawed overnight at 4^o^C and exoproteins were concentrated following the Trichloroacetic acid precipitation protocol described by Christie-Oleza et al., (2012)^1^. Protein pellets were dissolved in 60 μL Sample Buffer (Expedeon, UK) containing the RunBlue^©^ DTT Reducer (Expedeon, UK) following the manufacturer’s guidelines. Exoproteomes were visualised using SDS-PAGE (Expedeon, UK) following the manufacturer’s recommended settings. For protein identification a short run (~2 min) was performed to create a single gel band containing the entire exoproteome, as previously described by Christie-Oleza et al., (2012). In-gel reduction was performed prior to trypsin digestion and subsequent clean up as previously described by^1^. Samples were analysed by means of nanoLC‐ESI‐MS/MS using an Ultimate 3000 LC system (Dionex‐LC Packings) coupled to an Orbitrap Fusion mass spectrometer (Thermo Scientific, USA) using a 60 min LC separation on a 25 cm column and settings as previously described^2^. Quantification, statistical analyses and data visualisation of exoproteomes was carried out in Perseus^3^. The mass spectrometry proteomics data have been deposited in the ProteomeXchange Consortium via the PRoteomics IDEntifications (PRIDE) partner repository with the dataset identifier PXD014380 and 10.6019/PXD014380.

**Protein fractionation of the soluble *F. johnsoniae* cellular proteome**

To extract and enrich/purify proteins with phosphatase activity 500 ml of culture was grown. To obtain whole cell protein extract, cells were pelleted at 12000 rpm, and resuspended in 4 ml loading buffer (20 mM Tris HCl buffer, pH7.4, containing 250 mM NaCl), prior to bursting via French Press. This resulting protein milieu was centrifuged at 25000 rpm for 20 min at 4^o^C using a Beckman-Coulter JA25.50 fixed angle rotor to remove any residual cellular debris. Size-exclusion chromatography was used to isolate protein fractions with phosphatase activity. Both size-exclusion and anion-exchange chromatography (SEC and AIEC respectively) were performed using an ÄKTAPURIFIER UPC 10 (Cytiva). SEC used a HiLoad 16/600 Superdex 200 pg column (GE Healthcare) with 120 mL bed volume. Flow rate was 0.5 ml/min for four hours (1x column volume), using the loading buffer as an isocratic buffer. 1ml fractions were collected between 40-120 ml, and phosphatase activity was assayed using PNPP degradation and A405 absorption in a Wallac Victor 96-well plate reader. Fractions making up the two definite phosphatase-active peaks (peak 1 and peak 2, see Fig S6) were taken forward for further purification efforts.

The inherent property of phosphatases to bind the phosphonate moiety, which they cannot cleave was utilised, to further purify phosphatases from the active fractions following SEC. 5 ml EconoColumn (BioRad) gravity columns were loaded with 5 ml L-Histidyldiazobenzylphosphonic acid agarose (Sigma-Aldrich). Briefly, the column was equilibrated with 5x volume loading buffer, protein was loaded onto the column and left to bind for 1 hour. The column was then washed with 5x volume loading buffer to remove unbound protein, and bound protein was then eluted in 1x volume elution buffer 1 (loading buffer + 1M NaPO_4_). For peak 2, phosphatase activity of fractions was again assayed using PNPP degradation. Peak 1 failed to fractionate correctly using this method.

For peak 1, AIEC used a Mono Q 10/100 GL (Sigma-Aldrich) with 8 ml bed volume. Flow rate was 2 ml/min. Protein in loading buffer was loaded onto the column, then the column was washed with 5x volume loading buffer before a 0-50% gradient of elution buffer 2 (20 mM Tris HCl pH7.4, 1M NaCl) was applied over 40 mins (10x volume). This 50% ratio was held for a further 1x column volume before residual protein was washed off with 100% elution buffer 2. Phosphatase activity was again assayed using PNPP degradation.

**References**

1. Christie-Oleza, J.A. & Armengaud, J. In-depth analysis of exoproteomes from marine bacteria by shotgun liquid chromatography-tandem mass spectrometry: the *Ruegeria pomeroyi* DSS-3 case study. *Marine Drugs* **8**, 2223-2239 (2010).

2. Christie-Oleza, J.A., Armengaud, J., Guerin, P. & Scanlan, D.J. Functional distinctness in the exoproteomes of marine *Synechococcus*. *Environmental Microbiology* **17**, 3781-3794 (2015).

3. Tyanova, S. *et al.* The Perseus computational platform for comprehensive analysis of (prote)omics data. *Nature Methods* **13**, 731 (2016).

4. Zhu, Y. *et al.* Genetic analyses unravel the crucial role of a horizontally acquired alginate lyase for brown algal biomass degradation by *Zobellia galactanivorans*. *Environmental Microbiology* **19**, 2164-2181 (2017).

5. Kovach, M.E. *et al.* Four new derivatives of the broad-host-range cloning vector pBBR1MCS, carrying different antibiotic-resistance cassettes. *Gene* **166**, 175-176 (1995).

6. Kumar, S., Stecher, G. & Tamura, K. MEGA7: Molecular Evolutionary Genetics Analysis version 7.0 for bigger datasets. *Molecular Biology and Evolution* (2016).

7. Kappelmann, L. *et al.* Polysaccharide utilization loci of North Sea Flavobacteria as basis for using SusC/D-protein expression for predicting major phytoplankton glycans. *The ISME Journal* **13**, 76-91 (2019).

**Supplementary Tables Legend** (see Supplementary tables excel file, )

Table S1. Transcriptomic analysis of nutrient-limited DSM2064

Table S2. Whole-cell proteomic analysis of nutrient-limited DSM2064

Table S3. Detailed exoproteomic analysis of DSM2064

Table S4. Proteomic analysis of DSM2064 fractionated cellular proteome with strong phosphatase activity (peak 1). Separation of the active fraction was performed using Anion-Exchange Chromatography

Table S5. Proteomic analysis of DSM2064 fractionated cellular proteome with strong phosphatase activity (peak 2). Separation of the active fraction was performed using phosphonate-affinity Chromatography

Table S6. Genome characteristics of the *Flavobacterium* isolates used in this study

Table S7. Detailed exoproteomic analysis of F52

Table S8. Detailed exoproteomic analysis of LOA-5

Table S9. Detailed exoproteomic analysis of OSR001

Table S10. Detailed exoproteomic analysis of OSR003

Table S11. Detailed exoproteomic analysis of OSR004

Table S12. Detailed exoproteomic analysis of OSR005

Table S13. Detailed exoproteomic analysis of TSA_1_4_3

Table S14. Comparative genomics of ~100 *Flavobacterium* strains

Table S15. Detailed taxon list of Bacteroidetes isolates (n=468)

Table S16. Corresponding amino acid sequences for all locus tags within the OSR001 genome

Table S17. Corresponding amino acid sequences for all locus tags within the OSR002 genome

Table S18. Corresponding amino acid sequences for all locus tags within the OSR003 genome

Table S19. Corresponding amino acid sequences for all locus tags within the OSR004 genome

Table S20. Corresponding amino acid sequences for all locus tags within the OSR005 genome

Table S21. Corresponding amino acid sequences for all locus tags within the TSA_1_4_3 genome

Table S22. Corresponding amino acid sequences for all locus tags within the LOA-5 genome




*I/II*

*Flavobacterium*

*Chryseobacterium*

Other Bacteroidetes

Bacillales

Actinobacteria

*Streptomyces*

*Chloroflexi*

Cyanobacteria

Alphaproteobacteria

Betaproteobacteria

Gammaproteobacteria

Epsilonproteobacteria

**Figure S4. Phylogenetic comparison of PhoX found across all bacterial taxa.** Tree topology and branch lengths were calculated by maximum likelihood using the WAG+I+G4 model of evolution for 277 amino acid sequences based on sites in lQ-TREE software. Trees were visualized and annotated using the Interactive Tree of Life (ITOL) online server. The blue truncated ring (I/II) represents PhoX clades I and II. Black squares represent structurally or genetically characterised PhoX homologs. Green circles represent PhoX homologs found in the *Flavobacterium* isolates used in this study. IMG accession numbers for each homologs are given at the end of each leaf.
